# Genome-Wide Identification, Evolution, and Expression Patterns of the Fructose-1,6-Bisphosphatase Gene Family in *Saccharum* Species

**DOI:** 10.3390/plants14152433

**Published:** 2025-08-06

**Authors:** Chunyan Tian, Xiuting Hua, Peifang Zhao, Chunjia Li, Xujuan Li, Hongbo Liu, Xinlong Liu

**Affiliations:** 1 State Key Laboratory for Tropical Crop Breeding, Kunming 650205, China; tcy@yaas.org.cn (C.T.); hnzpf@163.com (P.Z.); lcj@yaas.org.cn (C.L.); lixujuan2011@163.com (X.L.); liuhongbo1982@126.com (H.L.); 2 Yunnan Key Laboratory of Sugarcane Genetic Improvement, Sugarcane Research Institute, Yunnan Academy of Agricultural Sciences, Kaiyuan 661699, China; 3 State Key Laboratory for Conservation and Utilization of Subtropical Agro-Bioresources, Nanning 530004, China; xiutinghua@126.com; 4 Guangxi Key Laboratory of Sugarcane Biology, Guangxi University, Nanning 530004, China

**Keywords:** *FBP* gene family, genome-wide identification, expression patterns, functional divergence, *Saccharum spontaneum*, *Saccharum officinarum*

## Abstract

Fructose-1,6-bisphosphatase (FBP) is a crucial regulatory enzyme in sucrose synthesis and photosynthetic carbon assimilation, functioning through two distinct isoforms: cytosolic FBP (cyFBP) and chloroplastic FBP (cpFBP). However, the identification and functional characterization of *FBP* genes in *Saccharum* remains limited. In this study, we conducted a systematic identification and comparative genomics analyses of *FBPs* in three *Saccharum* species. We further examined their expression patterns across leaf developmental zones, spatiotemporal profiles, and responses to diurnal rhythms and hormonal treatments. Our analysis identified 95 *FBP* genes, including 44 *cyFBPs* and 51 *cpFBPs*. Comparative analyses revealed significant divergence in physicochemical properties, gene structures, and motif compositions between the two isoforms. Expression profiling indicated that both *cyFBPs* and *cpFBPs* were predominantly expressed in leaves, particularly in maturing and mature zones. During diurnal cycles, their expression peaked around the night–day transition, with *cpFBPs* exhibiting earlier peaks than *cyFBPs*. *FBP genes* in *Saccharum spontaneum* displayed greater diurnal sensitivity than those in *Saccharum officinarum*. Hormonal treatments further revealed significant regulatory divergence in *FBP* genes, both between isoforms and across species. Notably, *cyFBP*_*2* and *cpFBP*_*2* members consistently exhibited higher expression levels across all datasets, suggesting their pivotal roles in sugarcane physiology. These findings not only identify potential target genes for enhancing sucrose accumulation, but also highlight the breeding value of *S. spontaneum* and *S. officinarum* in sugarcane breeding.

## 1. Introduction

Fructose-1,6-bisphosphatase (FBP, EC 3.1.3.11) is ubiquitously present across plant, animal, and microbial tissues, where it catalyzes the irreversible hydrolysis of fructose-1,6-bisphosphate (F-1,6-BP) to fructose-6-phosphate (F-6-P) and inorganic phosphate [1,2]. In higher plants, FBP exists as two distinct isoforms: a cytosolic form (cyFBP) and a chloroplastic form (cpFBP). The cyFBP catalyzes the production of F-6-P and serves as a key rate-limiting enzyme in the sucrose biosynthesis pathway [3,4]. Conversely, cpFBP participates in ribulose-1,5-bisphosphate (RuBP) regeneration in the Calvin cycle and contributes to the production of precursors for starch biosynthesis [5]. These two distinct isoforms establish FBP as a crucial metabolic regulator in plant sucrose synthesis and photosynthetic carbon metabolism. Moreover, cyFBP can be inhibited by fructose-2,6-bisphosphate (F-2,6-P_2_), adenosine monophosphate (AMP), Ca^2+^, and Mg^2+^; its activity is highest at a neutral pH in both plants and animals, whereas cpFBP is insensitive to F-2,6-P_2_ and AMP [6,7]. Sugarcane (*Saccharum* spp. hybrids), which uniquely stores sucrose in its stem vacuoles at exceptionally high concentrations, is an important model crop for studying C_4_ photosynthesis and carbohydrate metabolism [8]. However, the isoform-specific properties and functional roles of FBP in sugarcane remain to be systematically characterized. These knowledge gaps significantly hinder the effective utilization of cyFBP and cpFBP for the genetic improvement of sucrose-related traits in sugarcane breeding programs.

To date, *FBP* genes have been cloned from various species, such as *Spinacia oleracea*, *Triticum aestivum*, *Vigna radiata*, *Ricinus communis*, and other plants [9,10,11,12]. The research on functional analyses has revealed the critical roles of *FBP* genes in sucrose synthesis, carbon metabolism, photosynthetic rates, and stress responses. For example, the loss of *cyFBP* in rice significantly reduces the sucrose synthesis, resulting in severe growth retardation [13]. In transgenic *Arabidopsis thaliana*, the decreased expression of *cyFBP* limits sucrose biosynthesis, leading to the accumulation of phosphorylated intermediates [14]. Similarly, the transgenic potato plants with less than 20% of wild-type cyFBP activity show leaf accumulation of 3-phosphoglyceric acid, F-1,6-BP, and triose-phosphate (TP), accompanied by significant reductions in photosynthetic rates, sucrose synthesis, and starch accumulation [15]. Conversely, *cyFBP* overexpression enhances sucrose synthesis and promotes growth in *A. thaliana* [16] and tobacco (*Nicotiana tabacum*), where transgenic plants expressing *Brassica napus cyFBP* demonstrate increased photosynthetic rates, sucrose content, biomass, dry weight, and other yield-related traits [17]. For *cpFBP*, the expression of a cyanobacterial *cpFBP* in tobacco improved photosynthetic efficiency, CO_2_ fixation, and dry matter production compared with wild-type plants, along with increased levels of Calvin cycle intermediates and carbohydrate accumulation [18]. A similar result was observed in tomato, where *cpFBP* inhibition led to reduced fruit weight [19]. In contrast, *A. thaliana cpFBP* antisense plants unexpectedly showed increased leaf fresh weight and carbon assimilation rates [20], suggesting species-specific regulatory roles of *cpFBP* in photosynthetic metabolism. Interestingly, the expression of potato leaf *cpFBP* in tubers established an alternative starch biosynthesis pathway utilizing cytosolic TP, resulting in a higher starch content compared to wild-type tubers that rely solely on glucose-6-phosphate [21]. Furthermore, crucial roles of *cpFBPs* have been observed in high-temperature and drought stress responses in *Pyropia haitanensis* [22], fiber development in cotton (*Gossypium* spp.), and responses to Verticillium wilt and salt stress in cotton [23,24]. Collectively, these findings demonstrate that *cyFBP* genes play important roles in regulating sucrose biosynthesis, while *cpFBP* genes exhibit multifaceted roles in photosynthetic carbon assimilation, plant growth and development, as well as responses to biotic and abiotic stresses.

Sugarcane is the most important source of sugar and a promising crop for bioenergy production worldwide [25,26]. It accounts for approximately 80% of the global sucrose supply and contributes to about 40% of global ethanol production [27]. Theoretically, the maximum sucrose content in sugarcane stems can reach up to 30% of fresh weight [28,29]. Numerous studies have focused on sucrose synthesis, transport [30,31,32,33], and metabolism in sugarcane [34,35]. However, previous studies on sucrose synthesis in sugarcane have mainly focused on sucrose phosphate synthase (SPS) [36,37,38,39,40]. This highlights a novel strategy for enhancing sucrose accumulation through understanding and exploiting favorable *FBP* genes. Although the successful cloning of *cyFBP* in sugarcane was achieved in 2009 [41], a comprehensive characterization of the *FBP* gene family, including systematic identification, expression profiling, and functional analysis have yet to be conducted on sugarcane, mainly due to the unavailability of the sugarcane genome before the end of 2018 [41,42].

Modern sugarcane cultivars are highly polyploid and mainly derived from interspecific hybridizations between *S. officinarum* (2n = 80) and wild *S. spontaneum* (2n = 40–128) resource, with approximately 70–80% of their chromosomes originating from *S. officinarum*, 10–20% from *S. spontaneum*, and about 10% from interspecific recombination [43]. Considering the importance of *S. spontaneum* and *S. officinarum* in sugarcane breeding, we identified members of the *FBP* family by analyzing the genomes of *S. spontaneum* (AP85-441, 1n = 4x = 32), *S. officinarum* (LA-Purple, 2n = 8x = 80), and the *Saccharum* hybrid cultivar R570 (2n = 12x = 114) [44]. We performed a systematic analysis of their physicochemical characteristics, gene structure, conserved motifs, and promoter *cis*-acting regulatory elements. Furthermore, we investigated the phylogenetic relationships, gene duplication events, selection pressure, and collinearity to elucidate the evolutionary conservation and divergence of the *FBP* family. Notably, we examined the expression patterns of *cyFBPs* and *cpFBPs* across leaf developmental gradients, as well as their spatiotemporal expression dynamics during growth stages, diurnal rhythms, and phytohormone responses in the two *Saccharum* species. These analyses were based on publicly available RNA-seq datasets and experimental qRT-PCR validation. The findings presented here could provide (i) elite *FBP* gene resources for improving sucrose accumulation in sugarcane, (ii) a foundation for hormonal regulation of *FBP* expression, and (iii) scientific guidance for utilizing *S. spontaneum* and *S. officinarum* in sugarcane breeding programs.

## 2. Results

### 2.1. Identification and Physicochemical Properties of FBP Proteins in Saccharum

A total of 95 FBP proteins (Supplementary Table S1) were identified by HMM-based screening and conserved domain validation, comprising 17 in *S. spontaneum* (3.14 Gb), 44 in *S. officinarum* (7.42 Gb), and 34 in the hybrid cultivar R570 (5.04 Gb). The number of FBPs may be related to the genome size of the species. The physicochemical properties of FBPs were conserved across the three *Saccharum* species. The encoded proteins ranged from 237 to 516 amino acid residues (aa), with molecular weights (MWs) ranging from 25.72 to 55.56 kilodaltons (kDa) and an average of 39.16 kDa. The predicted isoelectric points (pIs) spanned from 5.17 to 8.90, with 96.8% (92/95) of FBPs having pI values below 7.0, indicating a bias toward acidic amino acids within this family. Approximately 40% (38/95) of FBPs were predicted to be unstable (instability index > 40). All FBPs showed negative grand average hydropathicity (GRAVY) values, suggesting their hydrophilic nature. Subcellular localization predictions indicated that 44 FBPs were cytoplasmic, whereas 51 were located in the chloroplast. Additionally, we observed significant variation between cpFBPs and cyFBPs: their size ranged from 237 to 343 aa with an average of 323.86 aa for cyFBPs, and 257 to 516 aa with an average of 392.43 aa for cpFBPs. The average MW of cpFBPs (39.61 kDa) was higher than that of cyFBPs (35.18 kDa), indicating a general trend of lower molecular weights in cyFBPs (Supplementary Table S1).

### 2.2. Phylogenetic Analysis of FBPs in Saccharum and Other Species

**Figure 1:**
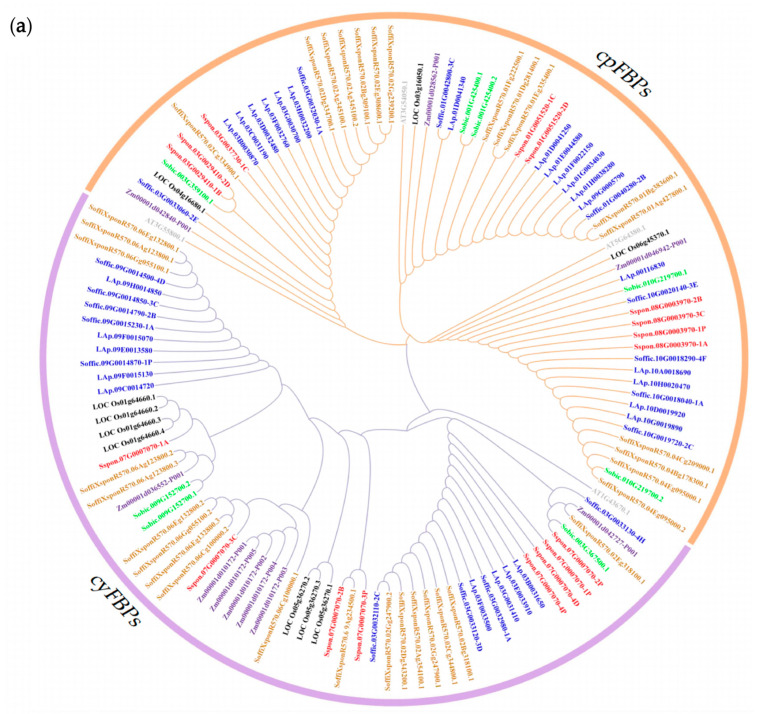

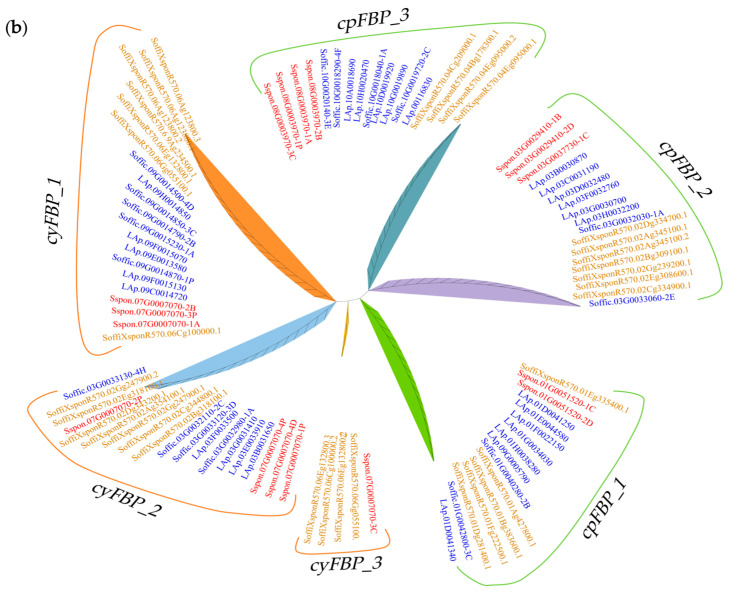
Phylogenetic tree of FBP proteins. (**a**) Phylogenetic tree of 127 *FBPs* from seven species. (**b**) Phylogenetic tree of 95 *FBPs* in three *Saccharum* species. Three *cyFBP* subgroups were designated as *cyFBP_1*, *cyFBP_2*, and *cyFBP_3*. Consistently, three *cpFBP* subgroups were designated as *cpFBP_1*, *cpFBP_2*, and *cpFBP_3*. ‘Sspon’ indicates *S. spontaneum* (AP85-441); ‘LAp’ and ‘Soffic’ indicate *S. officinarum* (LA-Purple); ‘SoffiXspon’ indicates hybrid R570; ‘LOC_Os’ indicates *O. sativa*; ‘Sobic’ indicates *S. bicolor*; ‘Zm’ indicates *Z. mays*; and ‘AT’ indicates *A. thaliana*. ‘*cyFBPs*’ and *‘cpFBPs*’ represent the cytosolic *FBP* genes and chloroplastic *FBP* genes, respectively. Gene labels with the same color represent *FBPs* from the same species.

To elucidate the evolutionary relationships of the *FBP* family, we identified 32 *FBPs* from four representative species: 8 from *Sorghum bicolor*, 10 from *Oryza sativa*, 10 from *Zea mays*, and 4 from *A. thaliana*. The 127 *FBPs* were classified into two groups (Figure 1a): *cpFBPs* (65 members) and *cyFBPs* (62 members). The results indicate that *S. officinarum* is more closely related to the hybrid R570 than to *S. spontaneum*. Furthermore, *Saccharum FBPs* exhibited a closer phylogenetic relationship with monocotyledonous species (*S. bicolor*, *Z. mays*, and *O. sativa*) than with the dicotyledonous *A. thaliana*. The number of *cpFBPs* exceeded that of *cyFBPs* in *S. officinarum*, *S. spontaneum*, *S. bicolor*, and *A. thaliana*, whereas *cyFBPs* were more abundant in R570, *Z. mays*, and *O. sativa*. Additionally, we constructed a phylogenetic tree using 95 *FBPs* from three *Saccharum* species, which were classified into six distinct subgroups: *cyFBP*_*1*, *cyFBP*_*2*, *cyFBP*_*3*, *cpFBP*_*1*, *cpFBP*_*2*, and *cpFBP*_*3* (Figure 1b).

### 2.3. Analysis of Gene Structure, Motif Distribution, and Conserved Domain of FBPs

To examine the structural composition of *FBP* genes in *Saccharum*, we constructed gene structure maps for *FBPs* in *S. spontaneum*, *S. officinarum*, and the hybrid cultivar R570 (Figure S1). Comparative analysis of exon–intron organization revealed distinct differences between *cyFBPs* and *cpFBPs*. The number of exons varied from 1 to 13, with *cyFBPs* containing 1–13 exons and *cpFBPs* containing 2–8 exons. Notably, two *cyFBP* genes (Sspon.07G0007070-3C and LAp.09E0013580) exhibited distinct exon patterns. Our results indicate that *cyFBPs* generally contains more exons than *cpFBPs* in three *Saccharum* species (Supplementary Tables S1 and S2), suggesting that they have more complex gene structures. Moreover, *FBPs* within the same subgroup exhibited highly conserved structure, consistent with their close phylogenetic relationships.

Analysis of *FBP* genes identified 10 conserved motifs across the three *Saccharum* species, with most genes containing 8–10 motifs. However, motif distribution patterns differed not only between *cpFBPs* and *cyFBPs*, but also among the three species. In *S. spontaneum*, *cyFBPs* contained 4–8 motifs, whereas *cpFBPs* contained 2–8 motifs, with motifs 8 and 10 unique to *cpFBPs*. Similarly, in *S. officinarum*, *cyFBPs* contained 8–9 motifs compared to 6–10 motifs in *cpFBPs*, with motif 9 exclusively present in certain *cpFBPs* but absent in *cyFBPs*. In the hybrid cultivar R570, *cyFBPs* contained 6–9 motifs, whereas all *cpFBPs* consistently contained 10 motifs, with motif 10 exclusively present in *cpFBPs*. Notably, the motif pattern map revealed that each phylogenetic subgroup exhibited a highly conserved motif arrangement and composition (Supplementary Figure S1), consistent with the phylogenetic and gene structure analyses.

Protein domain analysis showed that the *FBP* family contains two conserved domains: the N-terminal domain (FBPase_N) and the C-terminal domain (FBPase_C). All identified *FBPs* contained both domains, confirming their classification as members of the *FBP* family. Notably, *cpFBPs* contained additional sequences upstream of the N-terminal domain, a feature absent in *cyFBPs*.

### 2.4. Chromosomal Localization and Duplication Events of FBPs in Saccharum

**Figure 2:**
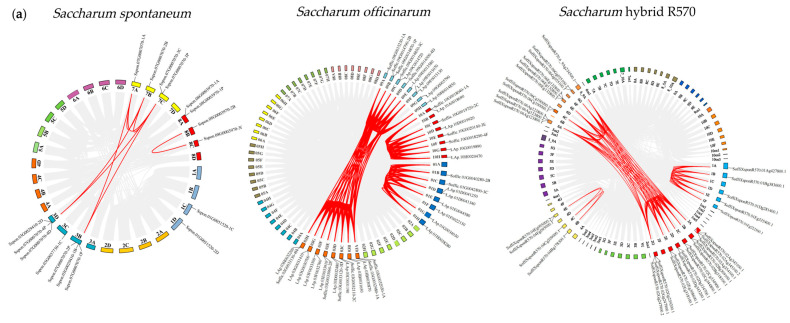

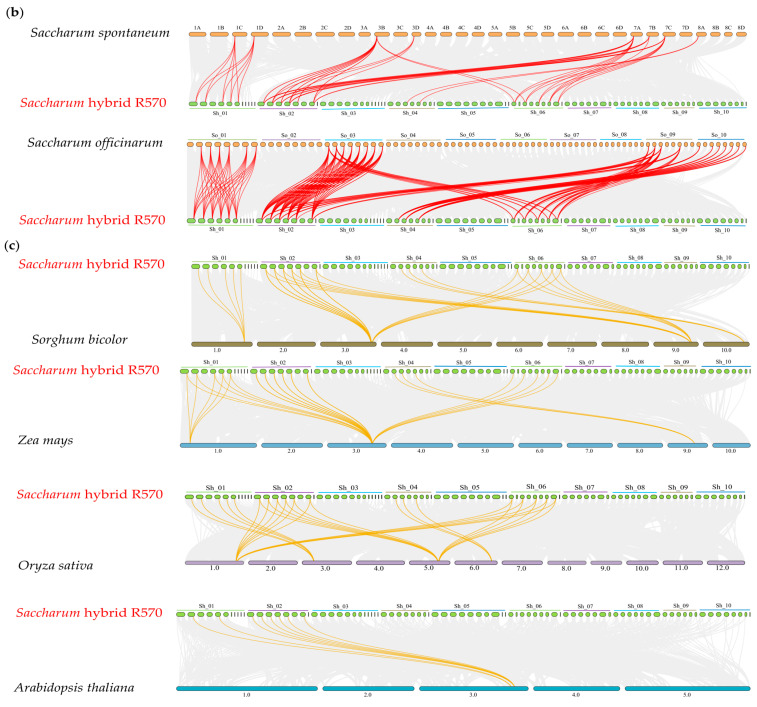
Chromosome mapping and collinearity analysis of *FBPs*. (**a**) Chromosome mapping and collinearity of *FBPs* in three *Saccharum* species. The chromosome number is indicated near each chromosome. Red lines represent syntenic *FBP* gene pairs, and gray lines are collinear blocks in the genome. The identified *FBP* genes are shown on the corresponding chromosomes. (**b**) Syntenic relationships between the hybrid R570, *S. spontaneum*, and *S. officinarum*. The chromosome number is indicated at the top or bottom of the chromosome. ‘*So*’ represents *S. officinarum*, and ‘*Sh*’ represents *Saccharum* hybrid R570. (**c**) Syntenic maps between R570 and other plant species, including *S. bicolor*, *Z. mays*, *O. sativa*, and *A. thaliana*. Brown–yellow lines represent syntenic *FBP* gene pairs, and gray lines are collinear blocks in the genome.

Chromosome mapping revealed an uneven distribution of *FBPs* across these three *Saccharum* genomes (Figure 2a). In *S. spontaneum*, 17 *FBPs* were dispersed across 11 chromosomes: 1C, 1D, 3B, 3C, 3D, 7A, 7B, 7C, 8A, 8B, and 8C. In *S. officinarum*, 43 *FBPs* were identified on 30 out of its 80 chromosomes, with one unplaced scaffold. Similarly, in R570, 34 *FBPs* were mapped to only 19 (22.1%) of its 86 chromosomes.

Gene duplication is a crucial mechanism underlying gene amplification and the evolution of novel functions. Typically, tandem and segmental duplication events are considered major drivers of gene family formation and whole-genome evolution [45,46,47]. In this study, we analyzed gene duplication events in three *Saccharum* species and observed numerous segmental duplications, along with dispersed and proximal events (Table S3). Notably, no tandem duplication events were detected. Specifically, *S. spontaneum* exhibited 9 segmental and 8 dispersed duplication events; *S. officinarum* contained 41 segmental, 2 dispersed, and 1 singleton event; while R570 showed 19 segmental, 8 proximal, 5 dispersed, and 2 transposed duplication events. These findings suggest that segmental duplication likely played a significant role in the expansion of the *FBP* family in *Saccharum*.

### 2.5. Collinearity Analysis of FBPs in Different Species

To further elucidate the evolutionary relationships of *FBPs* in *Saccharum* and other species, we performed synteny analysis between *S. spontaneum*, *S. officinarum*, and R570, as well as between R570 and three monocots (*S. bicolor*, *Z. mays*, and *O. sativa*) and one dicot (*A. thaliana*). We identified 206 syntenic gene pairs (137 orthologous) between *S. officinarum* and R570, compared to only 53 syntenic gene pairs (37 orthologous) between *S. spontaneum* and R570 (Figure 2b, Supplementary Table S4). These results suggest stronger syntenic collinearity between *S. officinarum* and R570 than between *S. spontaneum* and R570. The synteny maps between R570 and other species revealed strong collinearity with monocots (Figure 2c). *S. bicolor* had the highest number of homologs (36), followed by *Z. mays* (31) and *O. sativa* (30). In contrast, R570 shared fewer *FBP* homologs with *A. thaliana*. These findings further support the close evolutionary relationship among *Saccharum*, *S. bicolor*, and *Z. mays*.

### 2.6. Analysis of the Selection Pressure of FBPs in Saccharum

**Figure 3:**
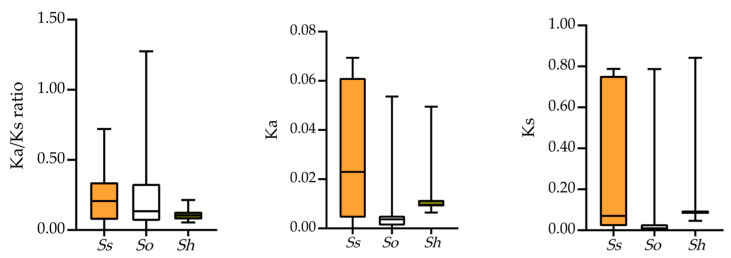
Comparisons of Ka/Ks, Ka, and Ks values of *FBP* pairs in three *Saccharum* species. *X*-axis shows the three *Saccharum* species, whereas the *Y*-axis shows the Ka/Ks ratio, Ka, and Ks. *Ss*, *So*, and *Sh* represent *S. spontaneum*, *S. officinarum*, and the *Saccharum* hybrid R570, respectively.

The calculation of Ka/Ks ratios is a valuable method for assessing sequence variations in protein orthologs across different species or taxa with unknown evolutionary histories [48]. In this study, we calculated the Ka/Ks ratios for orthologs and visualized them using box plots (Figure 3). The results show that all ortholog *FBP* pairs between *S. officinarum*, *S. spontaneum*, and R570 have Ka/Ks ratios < 1 (Supplementary Table S4), indicating purifying selection. A similar trend (Ka/Ks < 1) was observed between R570 and *S. bicolor*. These findings suggest that *FBPs* in *Saccharum* are under strong purifying selection.

### 2.7. Analysis of cis-Acting Elements of FBPs Promoter

We identified over 40 *cis*-acting elements, which were classified into four functional categories: light response, phytohormone response, stress response, and plant growth/metabolism regulation (Table 1). Light-responsive elements were the most abundant, and all *Saccharum FBPs* contained at least one such element. Within the phytohormone regulatory category, we specifically identified elements for abscisic acid response (ABRE), auxin, salicylic acid, and methyl jasmonate (MeJA). Stress-responsive elements included three major types: anaerobic response element (ARE), MYB-binding site involved in drought response (MBS), and low-temperature response (LTR). Additionally, we found elements regulating plant growth and development, such as those involved in zein metabolism, seed-specific regulation, meristem expression, and circadian control.

### 2.8. Expression Patterns of FBPs in Different Leaf Segments and Developmental Stages in S. spontaneum and S. officinarum

**Figure 4:**
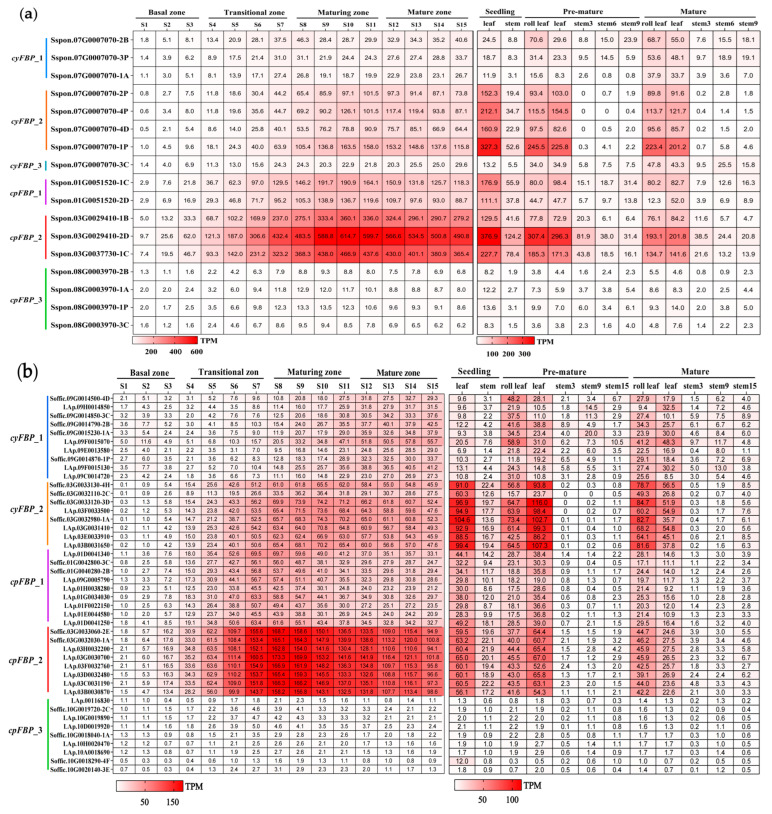
Expression patterns of *FBPs* in different leaf segments and three stages. (**a**) *S. spontaneum.* (**b**) *S. officinarum.* ‘S1-S15’ represents the 15 segments from the base to the tip of the leaf. Stem3, 6, and 9 for *S. spontaneum* and stem3, 9, and 15 for *S. officinarum* represent the immature, maturing, and mature stems, respectively. TPM represents transcripts per million. Six subgroups of the *FBP* family in *Saccharum* species are indicated on the left of the gene ID.

*FBPs* play crucial roles in sucrose synthesis, photosynthetic carbon assimilation, and partitioning in plant source organs. To investigate their contributions during leaf segmental development, we analyzed their expression patterns. The results reveal that both *cyFBPs* and *cpFBPs* are highly expressed in the high-photosynthetic regions of leaves (S6-S15 segments), particularly in the S7-S13 segments (Figure 4), while their expression levels are extremely low in the leaf base. Moreover, both *S. spontaneum* and *S. officinarum* exhibited higher expression in *cyFBP*_*2*, *cpFBP*_*2*, and *cpFBP*_*1* subgroup members, whereas the other subgroup members showed minimal expression. However, the expression levels in *S. spontaneum* followed the order *cpFBP*_*2* > *cpFBP*_*1* > *cyFBP*_*2*, while in *S. officinarum*, the pattern was *cpFBP*_*2* > *cyFBP*_*2* > *cpFBP*_*1*.

We further analyzed spatiotemporal expression patterns covering two tissues (leaf and stem) at the seedling stage and five tissues (roll leaf, mature leaf, stem3, stem6/9, and stem9/15) at the pre-mature and mature stages. The results reveal that both *cyFBPs* and *cpFBPs* exhibit consistently high expressions in leaves across different developmental stages, but show low expression in all tested stem tissues. Similarly, the *cyFBP*_*2*, *cpFBP*_*2*, and *cpFBP*_*1* subgroup members maintained higher expression levels than other subgroups (Figure 4), consistent with the observations in different leaf segments. However, the expression patterns of these subgroup members differed between the two species: in *S. spontaneum* (Figure 4a), the expression levels followed the order seedling stage > pre-mature stage > mature stage, while in *S. officinarum* (Figure 4b), the order for the majority of members was pre-mature stage > seedling stage > mature stage.

### 2.9. Expression Changes in FBPs During the Diurnal Rhythm of S. spontaneum and S. officinarum

**Figure 5:**
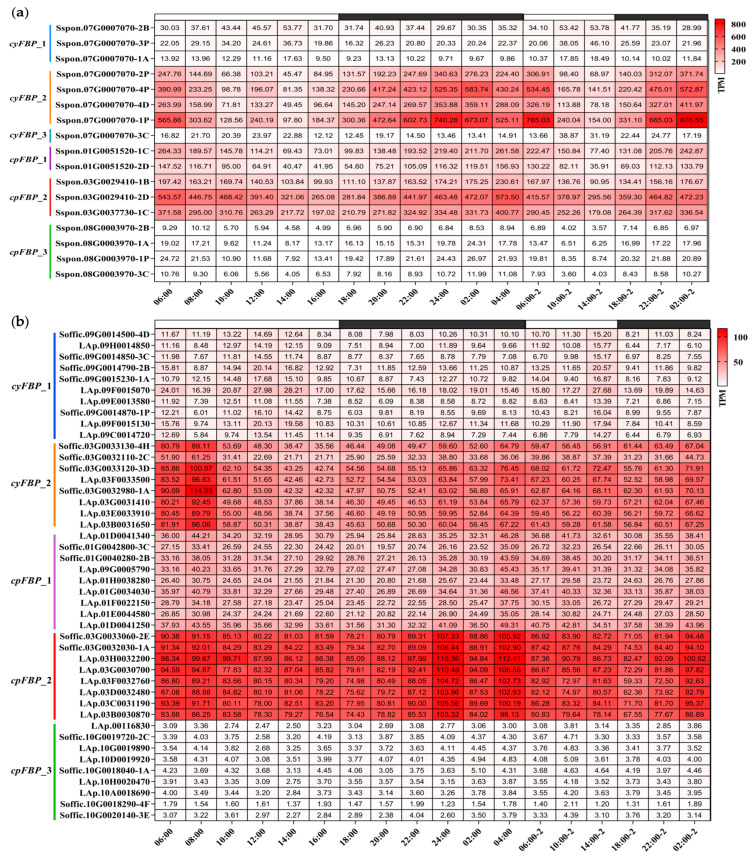

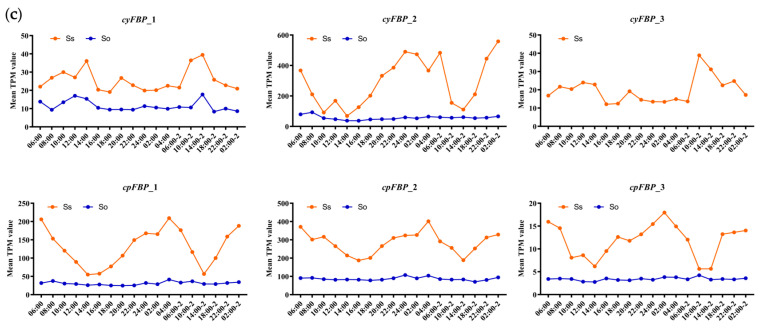
Expression patterns of *FBPs* at different time periods based on the TPM value. (**a**) *S. spontaneum.* Six subgroups of the *FBP* family in *Saccharum* species are indicated on the left of the gene ID. The white boxes at the top of the heatmaps represent light periods, while the black boxes represent dark periods. The corresponding time points are indicated at the bottom of the heatmap. (**b**) *S. officinarum.* (**c**) The expression trendlines of each subgroup in *S. Spontaneum* and *S. officinarum* during diurnal cycles. ‘*Ss*’ and ‘*So*’ represent *S. spontaneum and S. officinarum*, respectively. The mean TPM values are shown on the *Y*-axis, representing the average TPM values of all *FBP* members within each subgroup. The corresponding time points are displayed on the *X*-axis.

To characterize their diurnal expression patterns, we analyzed circadian transcriptome data, which included measurements at 2 h intervals for the first 24 h followed by 4 h sampling for an additional 24 h period. Heatmaps were generated to visualize the expression dynamics of *FBPs* in *S. spontaneum* (Figure 5a) and *S. officinarum* (Figure 5b) throughout the diurnal cycle. Temporal expression trends were further illustrated by plotting average TPM values for each subgroup (Figure 5c). Consistent with our previous findings, the *cyFBP*_*2*, *cpFBP*_*1*, and *cpFBP*_*2* subgroup members maintained consistently high expression levels in both species during the entire monitoring period. In *S. spontaneum*, the expression patterns of these *FBPs* were nearly identical across the diurnal cycle: expression levels decreased from early morning (06:00) to afternoon (14:00) and subsequently increased from 16:00 to the following morning (04:00). Peak expression for *cyFBP*_*2* occurred at 02:00 in the second cycle, while *cpFBPs* peaked earlier at 04:00 in the first cycle, with both subgroups showing minimal expression at 14:00. However, we observed that the expression levels of *FBPs* in *S. officinarum* did not show significant variance during diurnal cycles (Figure 5c), suggesting that *S. spontaneum* was more sensitive to diurnal rhythms. In addition, the expression for *cpFBP*_*2* members peaked at 24:00, whereas *cyFBP*_*2* members reached their highest levels at 08:00 in *S. officinarum*, which differed from those in *S. spontaneum*.

### 2.10. Expression Patterns of FBPs in Response to Hormone Treatments

**Figure 6:**
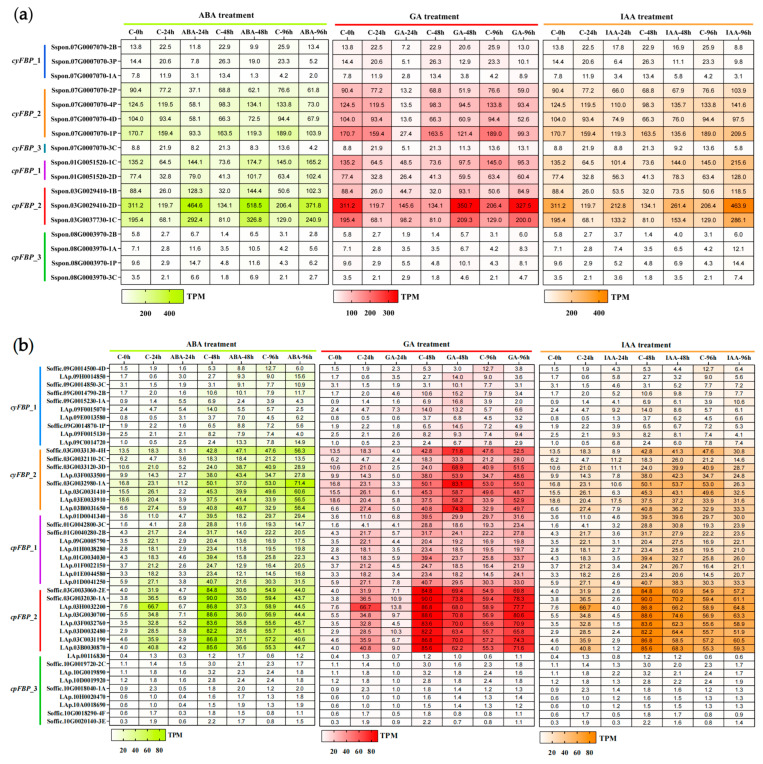
Expression patterns of *FBPs* under three hormone treatments in two *Saccharum* species. (**a**) *S. spontaneum.* (**b**) *S. officinarum.* The leaves from *S. spontaneum* and *S. officinarum* during the seedling stage were treated with ABA, GA, and IAA for 24 h, 48 h, and 96 h, respectively. The heatmaps from left to right represent ABA, GA, and IAA treatments, which are plotted in red and orange colors. ‘C’ represents the control.

To explore the hormone response mechanisms, we obtained RNA-seq datasets of *FBPs* from *S. spontaneum* and *S. officinarum* under ABA, GA, and IAA treatments from ScDB [49] and constructed corresponding heatmaps. Seedling leaves were collected at 0, 24, 48, and 96 h post-hormone treatment. The expression heatmaps revealed distinct expression patterns between *cpFBPs* and *cyFBPs* in both species following the three hormone treatments (Figure 6). In *S. spontaneum* (Figure 6a), *cpFBPs* were upregulated by all three hormones tested, with members of the *cpFBP_2* subgroup showing the greatest induction in response to the three hormone treatments. Most *cyFBPs*, however, exhibited significant downregulation compared to controls, with Sspon.07G0007070-1P exhibiting the strongest response to the three hormone treatments. In *S. officinarum* (Figure 6b), most *cpFBPs* displayed sustained downregulation by ABA at 96 h, an initial decrease at 48 h, followed by an increase at 96 h after GA and IAA treatments. Conversely, the majority of *cyFBPs* showed decreased expression at 24 h followed by an increase at 48–96 h under ABA and GA treatments, but decreased expression at 96 h under IAA treatment. Collectively, *FBPs*’ response to ABA, GA, and IAA treatments exhibited complex regulation, showing differences not only at the organelle level (between *cyFBPs* and *cpFBPs* within the same species) but also at the species level (between *S. spontaneum* and *S. officinarum*).

### 2.11. Expression Patterns of FBPs in Different Tissues of Two Sugarcane Cultivars During the Elongation Stage

**Figure 7:**
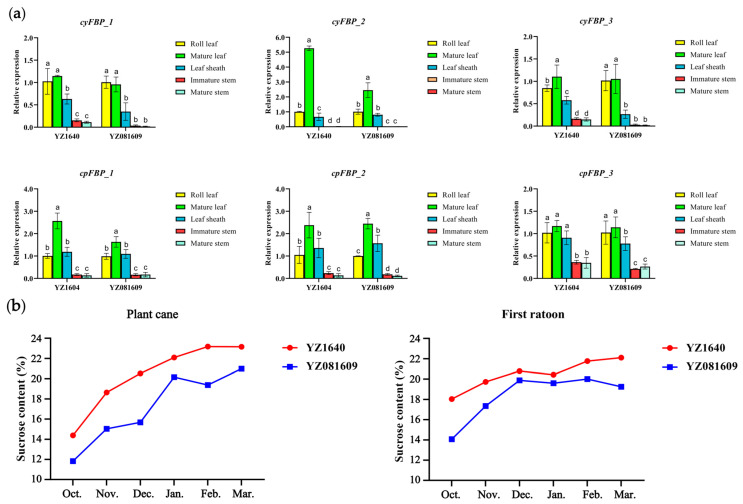
Detection of expression levels of *FBPs* and sucrose content in two sugarcane cultivars. (**a**) Detection of expression levels of *FBPs* across five tissues using qRT-PCR. The values on the *Y*-axis show the relative expression levels. The *X*-axis shows five tissues collected from the same clones of the cultivars. Different lowercase letters indicate a significant difference (*p* < 0.05), determined using one-way ANOVA with Tukey’s HSD post hoc test. *cyFBP*_*1*, *cyFBP*_*2*, *cyFBP*_*3*, *cpFBP*_*1*, *cpFBP*_*2*, and *cpFBP*_*3* indicate the name of detected subgroups, and the representative members in the hybrid R570 are listed in the brackets. (**b**) Comparison of sucrose content of two cultivars in plant and first ratoon cane. The sucrose content was continuously detected from October 2022 to March 2023 for plant cane and October 2023 to March 2024 for first ratoon cane.

To validate the expression of *FBPs* in different tissues, we analyzed two sugarcane cultivars/clones, YZ1640 and YZ081609, which exhibited differing sugar contents, with YZ1640 having a higher peak sucrose content than YZ081609 (Figure 7b). The relative expression levels of six *FBP* subgroups were detected across five tissues, namely roll leaf, mature leaf, leaf sheath, immature stem, and mature stem, during the elongation stage using qRT-PCR. The results demonstrate tissue-specific expression patterns of *FBPs*, with significantly higher expression levels in photosynthetic tissues (roll leaf, mature leaf, and leaf sheath), particularly in mature leaves, compared to substantially lower expression in stem tissues of sugarcane (Figure 7a). Similarly, the *FBPs* from the subgroups *cyFBP*_*2*, *cpFBP*_*1*, and *cpFBP*_*2* exhibited higher expression levels than those in other subgroups, consistent with the RNA-seq expression profiles. More interestingly, the expression levels of *cyFBPs* in the mature leaves of YZ1640 were higher than those in YZ081609, which may account for the higher sucrose synthesis capacity in YZ1640, although further validation is required.

## 3. Discussion

**Figure 8:**
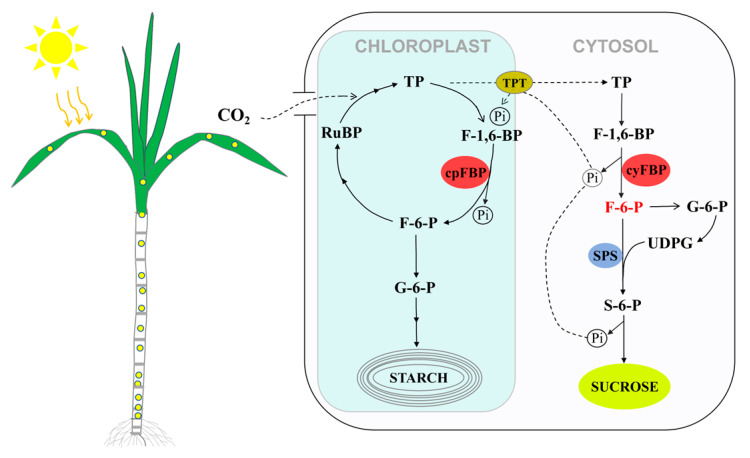
A simplified schematic for the physiological function of FBP in sucrose synthesis of sugarcane (modified from Daie, 1993 [3]). TP: triose phosphate; RuBP: ribulose bisphosphate; F-1,6-BP: fructose-1,6-bisphosphate; F-6-P: fructose-6-bisphosphate; Pi: inorganic phosphate, TPT: triose-P/phosphate translocator; S-6-P: sucrose-6-bisphosphate; G-6-P: glucose-6-bisphosphate; SPS: sucrose phosphate synthase.

Sugarcane is the most important sugar crop, supplying 80% of the global sugar demand. It is also a good model crop for studying sucrose accumulation and carbohydrate metabolism [30]. Previous studies have demonstrated that FBP plays crucial roles in photosynthetic carbon assimilation and sucrose biosynthesis through its two distinct isoforms [18,50,51,52]. As a typical C4 crop, sugarcane exhibits both high photosynthetic efficiency and sucrose accumulation capacity. Figure 8 presents a schematic model illustrating the involvement of cpFBP and cyFBP in the Calvin cycle and sucrose biosynthesis in sugarcane. The cpFBP participates in RuBP regeneration and starch biosynthesis in chloroplasts, processes that are closely associated with photosynthetic efficiency [5,18,20]. Subsequently, a portion of the photosynthetically derived triose phosphates is exported from chloroplasts to the cytosol, serving as precursors for sucrose biosynthesis. In the sucrose biosynthesis pathway (Figure 8), fructose-6-phosphate (F-6-P), produced by cyFBP catalysis, serves as a substrate for the production of uridine diphosphate glucose (UDPG). It further interacts with the synthesized UDPG to produce sucrose-6-phosphate, which is subsequently converted into sucrose. Therefore, we propose that F-6-P is a critical factor in sucrose biosynthesis. Modulating cyFBP activity to increase F-6-P levels may thus represent a promising strategy to enhance sucrose accumulation in sugarcane.

However, sugarcane is an extreme polyploid, which makes fundamental genetic studies on this plant more challenging than those conducted with diploid crops [43,53]. Our study identified 127 *FBPs* across three *Saccharum* species and four other representative species. Among them, *Saccharum* contains a greater number of *FBP* family members than diploid species, with 17 in *S. spontaneum*, 44 in *S. officinarum*, and 34 in the hybrid cultivar R570. Similar findings have been reported in cotton, where the tetraploid genomes of *G. barbadense* and *G. hirsutum* contain nearly double the number of *FBPs* compared with the diploid genomes of *G. arboreum* and *G. raimondii* [24]. This variation may be attributed to processes such as genome size, chromosome recombination, gene duplication, the differentiation of species and others [54,55]. Among the three analyzed *Saccharum* species, *S. officinarum* possesses both the largest genome and the highest gene count. These genomic features likely contributed to the greater number of *FBPs* identified in this species. According to the phylogenetic tree, 127 *FBPs* from seven species were categorized into two main groups, *cpFBPs* and *cyFBPs* (Figure 1a), consistent with the findings for cotton [24]. Moreover, we further divided the 95 *FBPs* from three *Saccharum* species into six subgroups to better understand their structures and motif compositions. The results reveal that *FBPs* within the same subgroup share highly conserved gene structures and motif composition (Supplementary Figure S1). This phenomenon has also been observed in other gene families [56,57,58]. However, the distribution of motifs differed not only between two *FBP* isoforms but also among the three *Saccharum* species (Figure S1), which may account for the functional distinction between *cyFBPs* and *cpFBPs* and species-specific differences.

Gene duplication is widely recognized as an important driver for gene family expansion [45]. After duplication, a gene may undergo several evolutionary outcomes, including degradation through functional mutations, preservation due to gene dosage effects, or sub-/neo-functionalization [59]. In this study, our analysis detected numerous WGD/segmental duplication events, suggesting that these mechanisms have been the primary drivers of *FBP* family expansion in *Saccharum*. Furthermore, we examined the collinearity of *FBPs* between the *Saccharum* hybrid R570 and three monocots (*S. bicolor*, *Z. mays*, and *O. sativa*) and one dicot (*A. thaliana*), and found many syntenic *FBP* pairs between *Saccharum* and the monocots. Notably, some *FBPs* in *Saccharum* do not have collinear counterparts in other species, implying that these members may have emerged after the divergence of sugarcane from related lineages via species-specific expansions, though further evidence is needed. For instance, nine *FBPs* in R570 have no collinear counterparts in *S. bicolor* (Supplementary Table S4). The amplification of *FBP* genes in *Saccharum* likely played a significant role in the species’ evolution. In addition, our findings suggest that the *FBP* family in *Saccharum* has primarily undergone purifying selection, as evidenced by the low Ka/Ks values of orthologous gene pairs [60].

These findings provide useful insights for the identification and evolution of the *FBP* gene family in sugarcane. However, modern sugarcane cultivars possess one of the most complex genomes among all crops, characterized by auto-/allo-polyploidy or aneuploidy and extreme ploidy levels ranging from 2n = 8x (octoploidy) to 2n = 12x (dodecaploidy). This complexity presents significant challenges for identifying and evolutionarily analyzing complex gene families in sugarcane. Major difficulties include (i) the large number of identified family members complicates accurate distinction between duplicated genes and alleles and (ii) diverse collinear relationships (one-to-one, one-to-many, and many-to-one) observed during genomic collinearity analysis. Consequently, the current methodologies for identifying complex gene families and inferring their evolutionary history in sugarcane may yield less robust results.

Studies on *FBP* genes in other plant species have demonstrated their involvement in various biological processes [16,23,24,51]. Since gene expression patterns are closely related to their functions in plants [61], we analyzed *FBP* expression profiles using four RNA-seq datasets and verified their tissue-specific expression in two sugarcane cultivars via qRT-PCR. Notably, all analyses indicated that members of the *cyFBP*_*2* and *cpFBP*_*2* subgroups consistently exhibited higher expression levels in *S. spontaneum*, *S. officinarum*, and sugarcane cultivars compared to other subgroups, positioning them as optimal candidates for genetic manipulation to enhance sucrose synthesis. Furthermore, both *cyFBPs* and *cpFBPs* showed elevated expression in maturing and mature leaves but low expression in stems, suggesting their functional importance in photosynthetic leaf tissue. Similar results were also found in another sucrose synthesis-related gene, *SPS*, with a higher expression in the leaf tissue than in stem tissue [39]. These findings align with the hypothesis that a high sucrose content in *Saccharum* stems results primarily from sucrose transport rather than in situ synthesis [39], a conclusion further supported by our data.

During diurnal cycles, *FBP* genes displayed similar expression patterns with obvious diurnal rhythms. Both *cyFBPs* and *cpFBPs* exhibited decreased levels from early morning to afternoon before increasing during the evening until the following dawn. However, we observed variations in their peak and trough expression times among different *cyFBP* and *cpFBP* members. For instance, in *S. spontaneum*, all four *cyFBP*_*2* members exhibited their lowest expression levels at 14:00, while peak expression times diverged: Sspon.07G0007070-1P and Sspon.07G0007070-4P peaked at 06:00, whereas Sspon.07G0007070-2P and Sspon.07G0007070-4D peaked earlier at 02:00 in the second cycle. In contrast, the three *cpFBP*_*2* members shared a common peak expression time at 04:00 in the first cycle but displayed divergent trough times. These results reveal that although *cyFBP* and *cpFBP* genes share consistent diurnal expression profiles, they show slight temporal differences in peak expression, with *cpFBP* genes peaking earlier than *cyFBP* genes. We hypothesize that this temporal difference may reflect distinct metabolic processes involving *cyFBPs* and *cpFBPs*. Specifically, the cyFBP-catalyzed reaction requires triose phosphates produced by the cpFBP-associated Calvin cycle as substrates, which might explain the delayed peak expression of *cyFBP* compared to *cpFBP*. Interestingly, both *cyFBP* and *cpFBP* expressions tend to peak at the end of the night or the start of the day, synchronizing with the expression of photosynthesis-related genes [62]. These findings suggest that (i) *cyFBP* and *cpFBP* may function coordinately to regulate sucrose biosynthesis in sugarcane; and (ii) these genes likely act as sugar-starvation responsive factors [8,63], with expression patterns synchronized with photosynthesis-related genes during diurnal cycles. However, the mechanisms underlying this coordinated regulation between *cyFBPs* and *cpFBPs* require further experimental validation.

Conversely, *cyFBPs* and *cpFBPs* showed distinct expression patterns in response to ABA, GA, and IAA treatments. In *S. spontaneum*, the expression levels of most *cyFBPs* decreased continuously from 24 to 96 h after treatment with ABA, GA, and IAA, whereas the expression levels of most *cpFBPs* increased steadily during the same period compared with the control. Moreover, this regulation was more complex in *S. officinarum*. In *S. officinarum*, the expression levels of most *cyFBPs* significantly decreased at 24 h and gradually increased from 48 to 96 h after treatment with ABA, GA, and IAA, whereas the expression levels of most *cpFBPs* decreased from 24 to 48 h and then increased at 96 h after treatment with GA and IAA. Under ABA treatment, however, the expression levels of *cpFBPs* decreased continuously from 24 to 96 h. These species-specific differential responses suggest the distinct functional specialization of *cyFBPs* and *cpFBPs* in hormone-mediated processes. Currently, the experimental evidence regarding the phytohormone-mediated regulation of the *FBP* gene family expression remains limited. This study provides a preliminary characterization of differential hormonal responsiveness between *cyFBP* and *cpFBP* genes, establishing a foundation for future research on manipulating sucrose metabolism in sugarcane through exogenous hormone application targeting these key enzymatic genes.

Furthermore, *S*. *spontaneum* and *S. officinarum* represent the two most crucial germplasm resources in sugarcane breeding, having contributed the origin of modern cultivars [64]. Significantly, *S. spontaneum* provides the primary genetic basis for stress tolerance traits, whereas *S. officinarum* serves as the major source of high sucrose content [65]. Previous studies by Nose et al. [66] and Jiang et al. [8] revealed that *S. spontaneum* generally exhibits higher photosynthetic rates than cultivated sugarcane, including *S. officinarum*. Our current findings indicate that *cyFBPs* and *cpFBPs* in *S. spontaneum* display significantly higher expression levels compared to those in *S. officinarum*. Considering that *cpFBPs* have been shown to influence photosynthetic rates in other plants [18,20], our results provide additional evidence supporting the higher photosynthetic rate of *S. spontaneum* relative to *S. officinarum*. Consequently, *S. spontaneum* can serve as a valuable germplasm resource for mining high-efficiency photosynthetic genes. However, whether *S. spontaneum* possesses a higher sucrose synthesis capability compared to *S. officinarum* requires further investigation. In this study, we detected higher expression levels of both *cyFBPs* and *cpFBPs* in YZ1640 compared to YZ081609, which showed a slightly positive correlation trend with stem sucrose content. Considering that high sucrose accumulation in sugarcane stems were mainly obtained through sucrose transport [39], we infer that high stem sucrose content may result from both high sucrose transport capacity and high sucrose synthesis capability in leaves. In addition, both *cyFBPs* and *cpFBPs* in *S. spontaneum* displayed strong diurnal expression oscillations with significant day–night variation, whereas *S. officinarum* showed minimal rhythmic expression changes. This differential response to diurnal cycles was consistently observed in core circadian clock and photorespiration-related genes, suggesting that the interspecies diurnal divergence may be attributed to sugar rather than light [8,62].

Additionally, distinct phytohormone response patterns were observed between *cyFBPs* and *cpFBPs*, as well as between the two *Saccharum* species. Specifically, in *S. spontaneum*, *cyFBPs* exhibited downregulation from 24 to 96 h under ABA and GA treatments. In contrast, *S. officinarum* showed a decreased expression at 24 h, followed by divergent expression patterns among *cyFBP* members at 48 h and 96 h. Meanwhile, *cpFBPs* also displayed significant differential expression patterns between the two species. In *S. spontaneum*, *cpFBPs* expression declined from 24 to 96 h compared to the control, whereas the opposite trend was observed in *S. officinarum* after ABA treatment. Similarly, expression differences of *cpFBPs* between the two species were detected under GA and IAA treatments. These results indicate interspecific divergence in the responses of *cyFBPs* and *cpFBPs* to hormonal treatments, highlighting the necessity of considering germplasm-specific variation when modulating sucrose metabolism via the hormone-mediated regulation of *FBP* genes in sugarcane.

## 4. Materials and Methods

### 4.1. Plant Materials

For qRT-PCR experiments, we used the sugarcane cultivar ‘YZ081609’and the promising new clone ‘YZ1640’ developed by the Sugarcane Research Institute of the Yunnan Academy of Agricultural Sciences [67]. Seed canes of both genotypes were planted in pots in February 2024. Five tissue samples, including roll leaves, mature leaves, leaf sheaths, as well as immature and mature stems, were collected seven months later during the elongation stage. The collected samples were rapidly frozen in liquid nitrogen and stored at −80 °C for subsequent analysis. Each sample was collected from three individual plants, representing three biological replicates.

### 4.2. Identification of FBP Family Members

The Hidden Markov Model (HMM) profiles corresponding to the FBP protein domains (PF00316 and PF18913) were obtained from the InterPro database (https://www.ebi.ac.uk/interpro/entry/pfam/PF00316/; https://www.ebi.ac.uk/interpro/entry/pfam/PF18913/, accessed on 20 August 2024). We then downloaded the genomes of *S. spontaneum* (AP85-441, 1n = 4x = 32) and *S. officinarum* (LA-Purple, 2n = 8x = 80) from the Sugarcane Genome Database (SGD, http://sugarcane.Zhangjisenlab.cn/sgd/html/download.html, accessed on 20 August 2024) and the *Saccharum* hybrid cultivar R570 (2n = 12x = 114) genome from the Sugarcane Genome Hub (https://sugarcane-genome.cirad.fr/, accessed on 14 August 2024). For comparative analysis, we obtained genome sequences of *S. bicolor* (v3.1.1), *Z. mays* (RefGen_V4), *O. sativa* (v7.0), and *A. thaliana* (TAIR10) from Phytozome (https://phytozome-next.jgi.doe.gov/, accessed on 17 July 2024).

The Simple HMM Search program in TBtools v2.119 [68] was performed to identify FBP protein sequences across these seven species. All candidate FBP proteins were further confirmed using the NCBI CD-Search database (https://www.ncbi.nlm.nih.gov/Structure/cdd/cdd.shtml, accessed on 16 September 2024) based on conserved domains analysis. The physicochemical properties of the identified FBP amino acid sequences were predicted using TBtools v2.119 [68]. Subcellular localization predictions were performed by Plant-PLoc (http://www.csbio.sjtu.edu.cn/bioinf/plant/, accessed on 16 September 2024).

### 4.3. Phylogenetic Analysis of the FBP Family

Multiple sequence alignment of the identified *FBPs* from *S. spontaneum*, *S. officinarum*, hybrid cultivar R570, *S. bicolor*, *O. sativa*, *Z. mays*, and *A. thaliana* was conducted using the ClustalW method in MEGA Ⅹ software [69]. Phylogenetic trees were constructed separately for seven species (including the three *Saccharum* species) using the maximum likelihood (ML) method with 1000 bootstrap replicates in MEGA Ⅹ [69]. The resulting phylogenetic trees were visualized and edited using the iTOL online platform (https://itol.embl.de/, accessed on 14 October 2024).

### 4.4. Visualization of Gene Structures, Motifs, and Protein Domains

Conserved motifs in FBP proteins from three *Saccharum* species were predicted by MEME (https://meme-suite.org/meme/tools/meme, accessed on 20 September 2024), with the maximum number of motifs set to 10 and other parameters set to default. The phylogenetic relationships, gene structures, motif distributions, and protein domains of *FBP* family members were then visualized and analyzed using the ‘Gene Structure View (Advanced)’ program in TBtools v2.119 [68].

### 4.5. Chromosomal Distribution, Collinearity, and Selection Pressure Analyses

Collinearity analysis was performed using the ‘One Step MCScanX-Super Fast’ function in TBtools v2.119 [68] with default parameters. Chromosome mapping and collinearity relationships were visualized using the ‘Advanced Circos’ function, while interspecific collinearity between the hybrid R570 and other species was displayed with the ‘Dual Synteny Plot’ function. The Ka/Ks ratios of orthologous gene pairs were calculated by TBtools v2.119 [68], with results visualized as box plots generated to investigate the distribution in GraphPad Prism 9.5 (San Diego, CA, USA). Generally, Ka/Ks ratios >1 indicate positive selection, ratios < 1 reflect purifying selection, and a ratio of 1 suggests neutral evolution (no selection pressure).

### 4.6. Cis-Acting Element Analysis

The 2 kb upstream sequences of identified *FBPs* were extracted as putative promoter regions using the ‘Gtf/Gff3 Sequences Extract’ program in TBtools v2.119 [68]. These sequences were then analyzed for *cis*-acting elements using the PlantCARE database (http://bioinformatics.psb.ugent.be/webtools/plantcare/html/, accessed on 26 September 2024).

### 4.7. Analysis of FBP Expression Profiling in S. spontaneum and S. officinarum

RNA-seq-based expression profiling datasets for leaf segmental development, different developmental stages, circadian rhythms, and hormone treatments were obtained from the ScDB (*Saccharum* Genome database, https://sugarcane.gxu.edu.cn/scdb/, accessed on 17 July 2024) [49]. Tissue sampling and experimental protocols were performed as described by Zhang et al. [30,31]. Specifically, leaves were divided into 15 segments from base to tip and further categorized into four zones. For hormonal response experiments, seedling-stage leaves of *S. spontaneum* and *S. officinarum* were treated with ABA, GA and IAA for 24, 48, and 96 h, respectively. Untreated leaves collected at corresponding time points served as control. Detailed experimental information is available in the ScDB database [49].

Based on these publicly available RNA-seq datasets, we generated heatmaps with GraphPad Prism 9.5 (San Diego, CA, USA). To further characterize diurnal expression patterns of different *FBP* subgroups in *S. spontaneum* and *S. officinarum*, we calculated the average TPM values for each subgroup and plotted the expression trends.

### 4.8. Validation of FBP Expression Levels in Sugarcane Using qRT-PCR

Firstly, tissue samples of two sugarcane cultivars were thoroughly ground in liquid nitrogen, and total RNA was extracted using the MolPure Plant RNA Kit (YeSen Biotechnology Co., Ltd., Shanghai, China). Secondly, the extracted RNA was verified through 1.0% agarose gel electrophoresis to assess its integrity. Finally, the obtained RNA was reverse-transcribed into cDNA following the instructions of the Evo M-MLV RT Mix Kit with gDNA Clean (Accurate Biology Co., Ltd., Hunan, China) for subsequent qRT-PCR analysis.

It is important to note that due to the high similarity of CDS sequences within each subgroup in *Saccharum*, it was nearly impossible to specifically detect individual *FBP* genes. Therefore, subgroup-specific primer pairs were designed for qRT-PCR analysis according to the representative R570 CDS sequences in each subgroup using Primer Premier 5.0 (Premier, PA, Canada). The qRT-PCR reaction system (20 µL) was prepared with the following components: 8.2 µL RNase free water, 1.0 µL of template cDNA, 0.4 µL each of forward and reverse primers, and 10.0 µL of 2×SYBR qPCR Master Mix (Accurate Biology Co., Ltd., Hunan, China). The qRT-PCR was performed by the QuantStudio 6 Flex real-time system (ABI, CA, USA). The reaction conditions were 95 °C for 30 s, followed by 40 cycles at 95 °C for 5 s, and 60 °C for 30 s, and the melting curves were analyzed after 40 cycles to confirm the PCR specificity. The *GAPDH* gene was used as the internal reference gene [70], and each sample was set with three biological replicates and three technical replicates. The relative expression levels of *FBPs* at the elongation stage of sugarcane plant growth was calculated using the 2^−∆∆Ct^ method [71]. All primer sequences used in this experiment are listed in Supplementary Table S5.

### 4.9. Detection of Sucrose Content in the Stems of Two Sugarcane Cultivars

To investigate the dynamics of sucrose content in ‘YZ081609’ and ‘YZ1640’, six healthy and representative stalks from each cultivar were collected from October 2022 to March 2023 for plant cane, and from October 2023 to March 2024 for first ratoon cane. Subsequently, the juice of the freshly collected six stalks was extracted using a cane juicer mechanical machine, and sucrose content (pol%) was measured by automatic saccharimeter, Autopol880 (Rudolph Research Analytical, NJ, USA).

### 4.10. Statistical Analysis

Statistical analyses were conducted using Excel 2010 for the statistics calculation of relevant data. Differences in *FBP* gene expression across five tissues in two sugarcane cultivars were analyzed by one-way ANOVA (*p* < 0.05) with LSD post hoc tests using SPSS Statistics 21.0, and the results were visualized as histograms using GraphPad Prism 9.5 (San Diego, USA).

## 5. Conclusions

In this study, we identified 95 *FBPs* (44 *cyFBPs* and 51 *cpFBPs*) from three *Saccharum* species, which were classified into six subgroups and shared a high degree of conservation in their gene structure, motif arrangement, and expression levels within each subgroup. Our results show that these *FBPs* are unevenly distributed across chromosomes and that WGD/segmental duplication is the major driving force for the expansion of this family. Both *cyFBP* and *cpFBP* members within the same subgroup exhibited high conservation in gene structure, motif arrangement, and expression patterns across *Saccharum* species. Expression profiling and qRT-PCR analyses revealed that both *cyFBPs* and *cpFBPs* exhibit predominant expression in leaf tissues. Furthermore, *cyFBPs* and *cpFBPs* showed more sensitive regulation by diurnal rhythm in *S. spontaneum* than in *S. officinarum*, suggesting that *S. spontaneum* may possess a higher photosynthetic rate. Additionally, we observed differential expression patterns in response to ABA, GA, and IAA treatments between *cyFBPs* and *cpFBPs*, as well as between the two *Saccharum* species. These findings highlight the need to consider the germplasm-specific variation when modulating sucrose metabolism via the hormone-mediated regulation of *FBP* genes in sugarcane. Notably, we identified that the *cyFBP*_*2* and *cpFBP*_*2* subgroup members could serve as optimal candidates for genetic manipulation to enhance sucrose synthesis in sugarcane. Our results provide a crucial theoretical foundation for additional research into the roles and mechanisms of *FBPs* and for their potential utilization to enhance sucrose accumulation in sugarcane.

## Figures and Tables

**Table 1:** List of *cis*-acting elements predicated of *FBPs* in three *Saccharum* species.

*Cis*-Element Name	Description	*S. spontaneum*		*S. officinarum*		Cultivar R570	
		Count	Genes	Count	Genes	Count	Genes
ABRE	abscisic acid responsiveness	82	16	198	37	293	34
AuxRR-core	auxin responsiveness	5	5	24	24	49	29
TGA-element	auxin responsiveness	15	8	49	34	40	26
GARE-motif	gibberellin responsiveness	3	3	9	3	10	7
P-box	gibberellin responsiveness	4	3	0	0	2	2
TATC-box	gibberellin responsiveness	3	2	14	3	12	5
TCA-element	salicylic acid responsiveness	9	5	13	7	19	10
CGTCA-motif	MeJA responsiveness	38	14	110	33	203	34
TGACG-motif	MeJA responsiveness	38	14	107	34	203	34
ARE	anaerobic induction	26	13	58	33	68	33
TC-rich repeats	defense and stress responsiveness	2	2	9	3	10	8
MBS	drought-inducibility	17	11	43	25	58	28
LTR	low-temperature responsiveness	14	11	24	21	18	15
GT1-motif	light responsiveness	2	2	7	5	19	10
Sp1	light responsiveness	35	11	66	22	71	19
G-box	light responsiveness	74	17	181	42	279	34
ACE	light responsiveness	1	1	2	2	4	3
MRE	light responsiveness	2	2	3	2	7	6
ATC-motif	light responsiveness	5	4	9	9	10	10
Box 4	light responsiveness	7	7	11	9	13	9
AE-box	light responsiveness	7	7	15	15	17	16
ATCT-motif	light responsiveness	1	1	3	3	2	2
GATA-motif	light responsiveness	12	11	37	29	51	28
TCCC-motif	light responsiveness	9	8	14	14	30	23
TCT-motif	light responsiveness	4	4	22	13	19	11
I-box	light responsiveness	4	2	18	12	39	23
L-box	light responsiveness	0	0	1	1	5	5
GA-motif	light responsiveness	2	2	7	7	4	4
3-AF1 binding	light responsiveness	0	0	3	3	3	3
chs-CMA2	light responsiveness	5	4	4	2	3	2
AT-rich element	DNA binding	2	2	8	8	6	6
CCAAT-box	MYBHv1 binding	26	13	70	32	66	27
O_2_-site	zein metabolism regulation	12	9	28	19	57	27
CAT-box	meristem expression	15	9	29	19	21	17
RY-element	seed-specific regulation	1	1	3	3	3	3
HD-Zip 1	palisade mesophyll cells	3	3	3	3	1	1
MBSI	flavonoid biosynthesis	0	0	3	3	1	1
circadian	circadian control	2	2	6	6	5	5
MSA-like	cell cycle regulation	5	5	12	8	11	7

## Data Availability

The complete *Saccharum* genome sequence information and RNA-seq-based expression profiling can be obtained from the ScDB website (https://sugarcane.gxu.edu.cn/scdb/, accessed on 19 June 2024). The datasets supporting the conclusions of this study are included in the article and its Supplementary Materials.

## Supplementary Materials

The following supporting information can be downloaded at: https://www.mdpi.com/article/10.3390/plants14152433/s1, Figure S1: Phylogenetic relationship, gene structure, motifs, and protein conserved domain of *FBPs* in three *Saccharum* species; Table S1: The detailed information of *FBP* genes identified in this study; Table S2: Analysis of exon numbers between *cyFBP* and *cpFBP* genes in three *Saccharum* species; Table S3: The gene type of *FBP* genes in three *Saccharum* species; Table S4: Orthologous relationships between *S. spontaneum*, *S.officinarum*, R570, and *S. bicolor*; Table S5: Primers used for qRT-PCR in this study.
