# Supplementary material for: Genome-Wide Identification, Evolution, and Expression Patterns of the Fructose-1,6-Bisphosphatase Gene Family in Saccharum Species
Source: Plants (Basel). 2025 Aug 6;14(15):2433. doi: 10.3390/plants14152433 (PMC12349412; doi:10.3390/plants14152433)
Supplement: Supplementary file 1 [file plants-14-02433-s001.zip › Revised Supplementary Figure S1.pdf]

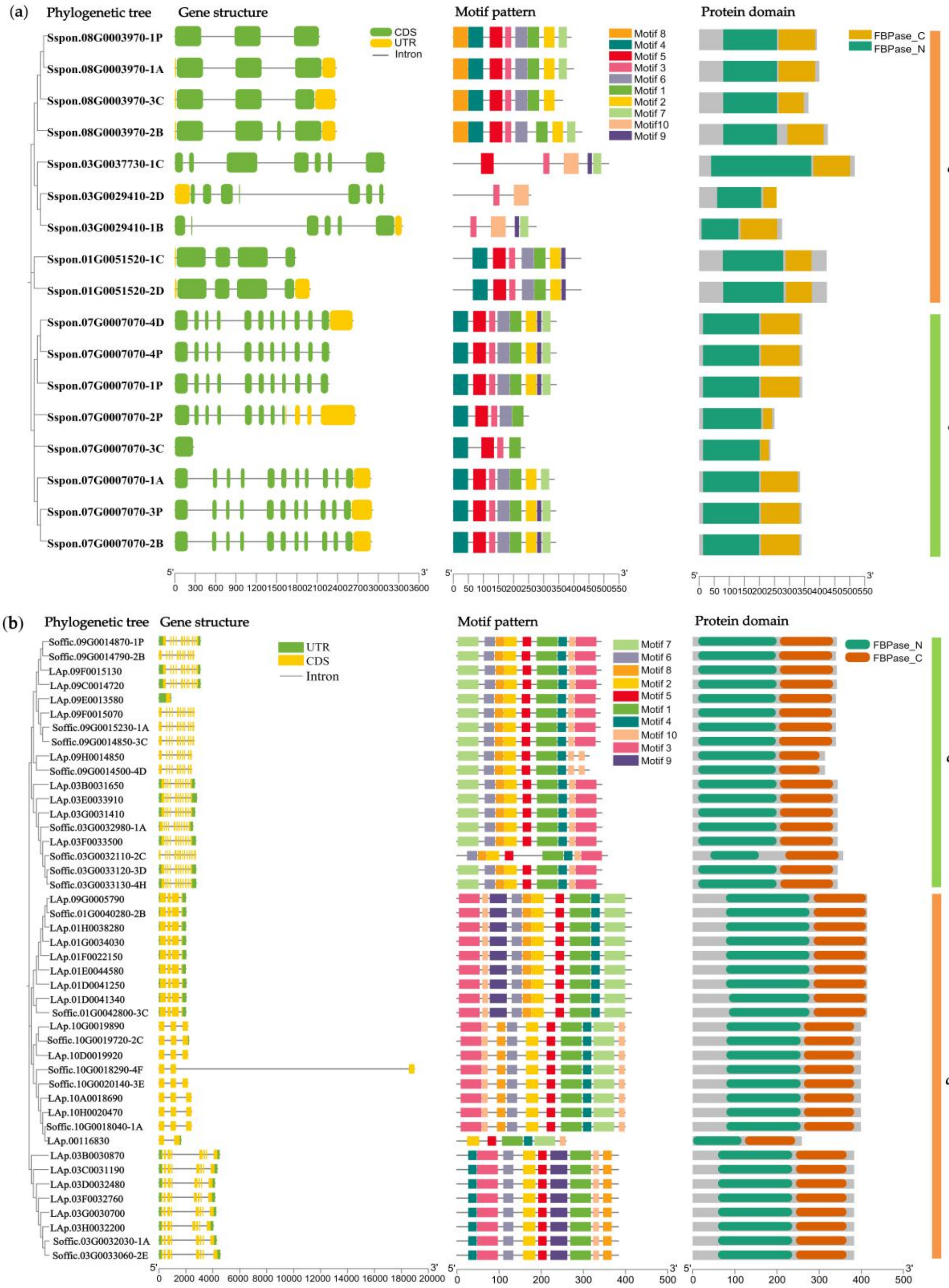

(a)
Phylogenetic tree
Gene structure
Motif pattern
Protein domain
CDS
UTR
Intron
Motif 8
Motif 4
Motif 5
Motif 3
Motif 6
Motif 1
Motif 2
Motif 7
Motif10
Motif 9
FBPase_C
FBPase_N
Sspon.08G0003970-1P
Sspon.08G0003970-1A
Sspon.08G0003970-3C
Sspon.08G0003970-2B
Sspon.03G0037730-1C
Sspon.03G0029410-2D
Sspon.03G0029410-1B
Sspon.01G0051520-1C
Sspon.01G0051520-2D
Sspon.07G0007070-4D
Sspon.07G0007070-4P
Sspon.07G0007070-1P
Sspon.07G0007070-2P
Sspon.07G0007070-3C
Sspon.07G0007070-1A
Sspon.07G0007070-3P
Sspon.07G0007070-2B
cpFBPs
cyFBPs
(b)
Phylogenetic tree
Gene structure
Motif pattern
Protein domain
UTR
CDS
Intron
Motif 7
Motif 6
Motif 8
Motif 2
Motif 5
Motif 1
Motif 4
Motif 10
Motif 3
Motif 9
FBPase_N
FBPase_C
Soffic.09G0014870-1P
Soffic.09G0014790-2B
LAp.09F0015130
LAp.09C0014720
LAp.09E0013580
LAp.09F0015070
Soffic.09G0015230-1A
Soffic.09G0014850-3C
LAp.09H0014850
Soffic.09G0014500-4D
LAp.03B0031650
LAp.03E0033910
LAp.03G0031410
Soffic.03G0032980-1A
LAp.03F0033500
Soffic.03G0032110-2C
Soffic.03G0033120-3D
Soffic.03G0033130-4H
LAp.09G0005790
Soffic.01G0040280-2B
LAp.01H0038280
LAp.01G0034030
LAp.01F0022150
LAp.01E0044580
LAp.01D0041250
LAp.01D0041340
Soffic.01G0042800-3C
LAp.10G0019890
Soffic.10G0019720-2C
LAp.10D0019920
Soffic.10G0018290-4F
Soffic.10G0020140-3E
LAp.10A0018690
LAp.10H0020470
Soffic.10G0018040-1A
LAp.00116830
LAp.03B0030870
LAp.03C0031190
LAp.03D0032480
LAp.03F0032760
LAp.03G0030700
LAp.03H0032200
Soffic.03G0032030-1A
Soffic.03G0033060-2E
cyFBPs
cpFBPs

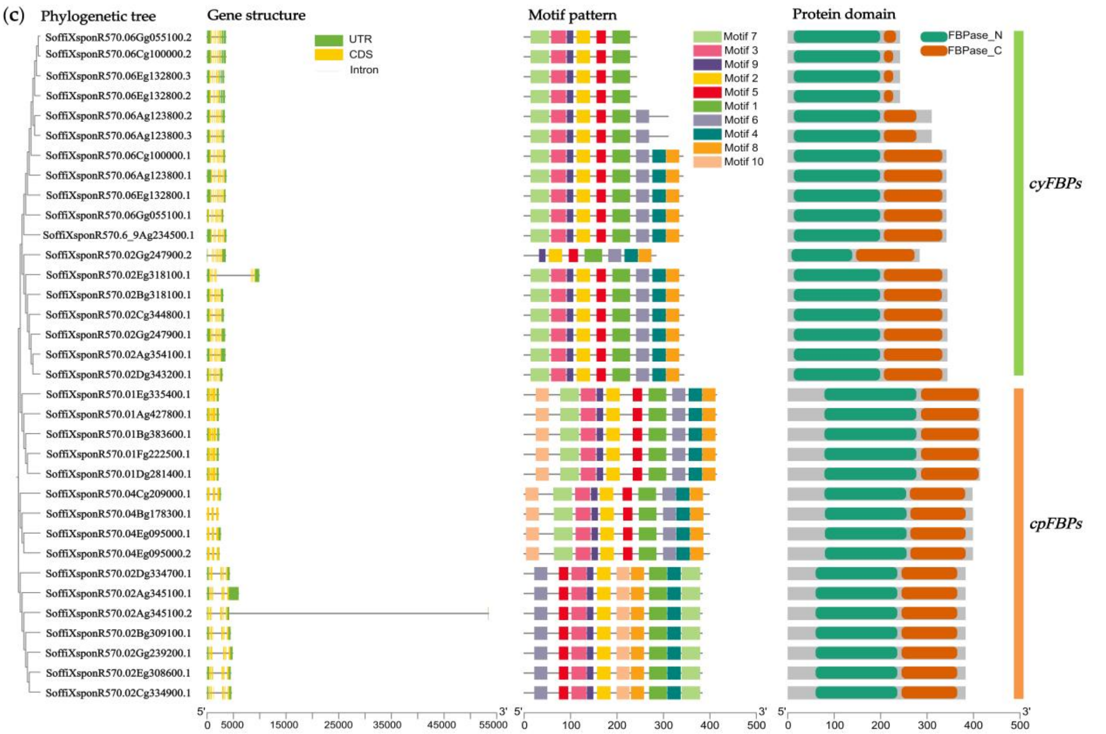

Figure S1. Phylogenetic relationship, gene structure, motifs, and protein conserved domain of *FBPs* in three *Saccharum* species. (**a**) *S. spontaneum*. (**b**) *S. officinarum*. (**c**) The *Saccharum* hybrid R570.
